# Expectations Among Patients and Health Professionals Regarding Web-Based Interventions for Depression in Primary Care: A Qualitative Study

**DOI:** 10.2196/jmir.3985

**Published:** 2015-03-10

**Authors:** Jesús Montero-Marín, Javier Prado-Abril, Cristina Botella, Fermin Mayoral-Cleries, Rosa Baños, Paola Herrera-Mercadal, Pablo Romero-Sanchiz, Margalida Gili, Adoración Castro, Raquel Nogueira, Javier García-Campayo

**Affiliations:** ^1^Faculty of Health and SportsUniversity of ZaragozaHuescaSpain; ^2^REDIAPP “Research Network on Preventative Activities and Health Promotion” (RD06/0018/0017)ZaragozaSpain; ^3^IIS Aragon: Health Research InstituteZaragozaSpain; ^4^Red de Excelencia PROMOSAM (PSI2014-56303-REDT). MINECOMadridSpain; ^5^Psychosocial Rehabilitation and Recovery CenterMental Health ServiceSanta Maria Hospital, GSS “Gestió de Serveis Sanitaris"LeridaSpain; ^6^Department of Basic PsychologyClinic and PsychobiologyUniversity Jaume ICastellonSpain; ^7^UGC Salud MentalHospital Regional UniversitarioInstituto de BiomedicinaMálagaSpain; ^8^CIBER Fisiopatología Obesidad y Nutrición (CIBERObn)Instituto Salud Carlos IIISantiago de CompostelaSpain; ^9^Department of Personality, Evaluation and Psychological TreatmentUniversity of ValenciaValenciaSpain; ^10^Institut Universitari d’Investigació en Ciències de la Salut (IUNICS)University of Balearic IslandsMallorcaSpain; ^11^Department of PersonalityAssesment and Psychological TreatmentUniversity of MálagaMálagaSpain; ^12^Psychiatry ServiceMiguel Servet University HospitalZaragozaSpain

**Keywords:** depression, computer-delivered psychotherapy, qualitative methods, expectations

## Abstract

**Background:**

One-quarter of the world’s population will suffer from depression symptoms at some point in their lives. Mental health services in developed countries are overburdened. Therefore, cost-effective interventions that provide mental health care solutions such as Web-based psychotherapy programs have been proposed.

**Objective:**

The intent of the study was to identify expectations regarding Web-based psychotherapy for the treatment of depression in primary care among patients and health professionals that might facilitate or hinder its effects.

**Methods:**

The expectations of untreated patients and health professionals were examined by means of interviews and focus groups. There were 43 participants (20 patients with mild and moderate levels of depression, 11 primary care physicians, and 12 managers; 22 of them for interviews and 21 for groups). A thematic content analysis from the grounded theory for interviews, and an analysis of the discursive positions of participants based on the sociological model for groups were performed. Interpretations were achieved by agreement between three independent analysts.

**Results:**

All participants showed a good general acceptance of Web-based psychotherapy, appreciating possible advantages and improvements. Patients, physicians, and managers shared the same conceptualization of their expectations, although highlighting different aspects. Patients focused on the need for individualized and personalized interaction, while professionals highlighted the need for the standardization of the program. Physicians were concerned with extra workload, while managers were worried about optimizing cost-effectiveness.

**Conclusions:**

Expectations of the different participants can conflict with each other. Finding a balanced position among them is needed if we are to harmoniously implement effective Web-based interventions for depression in routine clinical practice.

## Introduction

 Depression currently ranks fourth in disorders with the highest disease burden, and in the coming five years, it will cause the second-highest level of disability worldwide [1-3]. It has been said that 25% of the population will experience depression symptoms at some point in their lives [4]. Depression brings with it significant economic, personal, inter-personal, and societal costs, and is associated with important impairments in quality of life and increased mortality [5-8].

Antidepressant medication is the most common form of treatment for depressive patients in primary care [9], but adherence is low [10,11]. In fact, two-thirds of depressed primary care patients would prefer psychological treatments to pharmacotherapy [12-17]. Psychological treatments obtain better results than pharmacological ones in adherence, relapse, recovery rates, and chronicity, reducing the number of physician visits and hospital days [18,19]. Despite their effectiveness, face-to-face psychotherapy interventions face some serious limitations. These include difficulties in delivering interventions to the community due to strong barriers in providing psychotherapies in current models of service delivery; constraints in health care resources; limited availability of clinicians, especially in rural areas; and low participation rates, even if access to those interventions is at little or no cost [20-23]. Mental health services in developed countries are overburdened, and given the lack of intended resources, are in need of cost-effective alternatives [24].

Attempts to overcome barriers to access have been addressed recently through the evolution of a new understanding in mental health care that recognizes low-intensity services, such as bibliotherapy, psychoeducation, and Web-based interventions [25]. Low-intensity interventions signify treatments that limit specialist time or use this time in a cost-effective manner and are provided, when appropriate, as a first option before referral to high-intensity interventions in which the presence of the therapist is higher [26]. For example, when offered by an integrated health care provider using a stepped-care model, it has been suggested that the first choice should be the least intensive intervention that is appropriate for a person, enabling people to step up or down the pathway according to changing needs and in response to treatment [18]. The Internet offers a new way of providing psychological treatment for common mental health problems such as depression [27,28], and may attract people who do not make use of traditional mental health services [23]. Web-based treatment has been described as an intervention that is operationalized and transformed for delivery via the Internet [29]. It is usually highly structured and can be roughly divided into unguided and guided interventions, with an identified therapist [27,28,30]. Guided Internet interventions are considered to be more effective in reducing depression symptoms than unguided ones [31,32], with average levels of adherence estimated at 26% in unguided interventions and 72% in guided interventions [33]. Web-based interventions may also resemble traditional psychotherapy with scheduled sessions [34], but a general characteristic is that patients are reached from a distance and that therapist time is reduced compared with face-to-face treatments [35].

Several studies show that Web-based psychological treatments are effective for the treatment of depression, in both unguided and guided interventions, although the effects are more favorable in the latter case [33,36-38]. It has even been said that Web-based treatments with clinician assistance as brief as 1 hour per patient can work as well as face-to-face therapy [39]. Web-based psychotherapy could be viable not only in the context of specialist services, but also at the primary care level [40,41], and could be particularly recommended as a first treatment step for self-help in treating depression before visiting a psychiatrist or psychologist [42]. However, in spite of its efficiency, there are some limitations to its systematic use. One of the main ones is that attrition rates could be substantial [28,43-45]. In most cases, patients withdraw for personal reasons, not because of problems with the technology or the social environment. Interestingly, these kinds of interventions are generally acceptable for both patients and professionals, although the attitudes of professionals toward this type of psychotherapy seem to be more negative than the attitudes of patients themselves [38,46,47].

Therefore, if we are to develop and deploy this kind of program, maximizing acceptance and using it as a part of public health services, as in this case, we must be sensitive to the different views and attitudes of all participants in the therapeutic process. There may be different types of expectations or requirements according to the perspective of each type of participant. Given this background, the aim of the present study was to identify expectations among both patients and health professionals that may serve as barriers or facilitators to engagement with treatment when performing Web-based psychotherapy for depression in primary care, in order to increase adherence and effectiveness, improving the care offered to these types of patients.

## Methods

A qualitative design was used to collect information from a wide range of purposefully and theoretically guided samples of depressed patients and health professionals. Both in-depth interviews and discussion groups were used to access the subjectivity and the processes involved in generating expectations [48]. In-depth interviews were carried out by a single interviewer and focus groups were moderated by an interviewer and an observer, both of them female psychologists and researchers, with previous experience in the field and no previous contact with participants.

Patients were recruited from the Spanish autonomous region of Aragon during their visits to primary care, and diagnosis was established using the MINI Neuropsychiatric Interview in its Spanish version [49]. Patients were selected when they reported mild or moderate depression symptoms according to cut-off points of the Beck Depression Inventory [50]. Family physicians and managers were chosen from the catchment area, based on whether their workplace was rural or urban, and contacted by telephone. Participants were selected with consideration given to the variables of age, gender, residential setting (“urban”, “rural”), and affinity for technology (by means of the question: what is your level of affinity for new technologies?, with “high”, “intermediate”, “low” as possible responses), in order to gather plentiful and varied information. Participants had no previous knowledge of the researchers or the project. No patients refused to participate in the interviews, although some of them did not attend the discussion groups. No physicians or managers refused to participate; however, it was difficult to establish meeting dates because of their work schedule. Table 1 outlines the main characteristics of the 43 participants (20 patients, 11 physicians, 12 managers).

A standardized protocol was designed to guide the interviews and groups, including the preparation of a topic list (Table 2) to be addressed, with previously tested, open suggestions that could be of interest.

The objectives of the study were indirectly raised and questions asked about the topics in an open and progressive way. The interviewer and/or moderator were introduced to the participants as research psychologists and assumed a minimally orientative role, limiting their interventions to addressing the topics in the script. The setting for data collection was a neutral room in the hospital where the project was conducted, without the presence of non-participants. No individual or group interviews lasted more than 90 minutes. They were digitally audio-recorded and transcribed to obtain the final set of qualitative data for the analysis, which were revised by participants and added to the field notes made after interviews and groups. Participants provided written informed consent to participate in the study. There were no repeat interviews. This study forms part of a mixed-method research project that includes a randomized controlled trial with different participants [51]. The project was approved by the Regional Ethics Committee of Aragon, Spain.

A thematic content analysis was performed from grounded theory in order to develop and to define until saturation the emergent categories of analysis derived from the interview data [52-54]. Secondly, a sociological approach that analyzed discursive positions of participants in focal groups was used to complement the first approximation [55,56]. All the analyses were developed using Maxqda-2007 software, in an iterative way by agreement between the three researchers (JMM, JPA, JGC), and discussed with those in charge of the interviews and groups (PR, PHM) [56]. Moreover, participants provided their agreement with the interpretations. This methodological triangulation was able to increase consistency and rigor by combining multiple techniques, maximizing the breadth and depth of perspectives. The complete details of the study protocol are reported elsewhere [57].

**Table 1:** Characteristics of participants (n=43).^a^

Stratification variables		Interviews			Groups		
		Patients n=12	Physicians n=5	Managers n=5	Patients n=8	Physicians n=6	Managers n=7
**Age**							
	20-40 years	3 (7%)	2 (5%)	0 (0%)	2 (5%)	3 (7%)	1 (2%)
	41-60 years	6 (14%)	2 (5%)	5 (12%)	5 (12%)	2 (5%)	5 (12%)
	>60 years	3 (7%)	1 (2%)	0 (0%)	1 (2%)	1 (2%)	1 (2%)
**Sex**							
	Male	5 (12%)	1 (2%)	4 (9%)	1 (2%)	2 (5%)	2 (5%)
	Female	7 (16%)	4 (9%)	1 (2%)	7 (16%)	4 (9%)	5 (12%)
**Residential setting**							
	Urban	9 (21%)	3 (7%)	4 (9%)	5 (12%)	4 (9%)	5 (12%)
	Rural	3 (7%)	2 (5%)	1 (2%)	3 (7%)	2 (5%)	2 (5%)
**Level of education**							
	Primary	4 (9%)	0 (0%)	0 (0%)	3 (7%)	0 (0%)	0 (0%)
	Secondary	5 (12%)	0 (0%)	0 (0%)	3 (7%)	0 (0%)	0 (0%)
	University	3 (7%)	5 (12%)	5 (12%)	2 (5%)	6 (14%)	7 (16%)
**Affinity for technology**							
	High	6 (14%)	2 (5%)	2 (5%)	3 (7%)	4 (9%)	5 (12%)
	Intermediate	3 (7%)	3 (7%)	3 (7%)	4 (9%)	2 (5%)	2 (5%)
	Low	3 (7%)	0 (0%)	0 (0%)	1 (2%)	0 (0%)	0 (0%)

^a^Out of the total of participating patients, 40% (17/43) had university studies and 60% (26/43) had high school education. Half of the patients (10/20) presented with mild symptoms of depression, and the other half (10/20) presented with moderate symptoms.

**Table 2:** Questions asked and topic list.

Questions asked	Topic list
**What is your relationship with technology? Have you received any training?**	
	Skills
	Resources
	Routine use
	Reasons
	Like / dislike
**Do you think new technologies can help to improve people’s lives?**	
	Relationship machine/man
	Face-to-face vs Web-based
	Support tool vs replacement tool
**What do you think about the use of Web-based therapy to support the actions of health professionals? And as further therapies?**	
	Possibilities of Web-based therapy
	Virtual and anonymous relationship
	Health professional vs program
**What about the possibility of offering Web-based therapy for depressive disorders? Do you see it as feasible?**	
	What would you ask/expect?
	Information
	Difficulties
	Aspects that could be helpful
	Process from primary care
	Professional support

## Results

**Figure 1:**
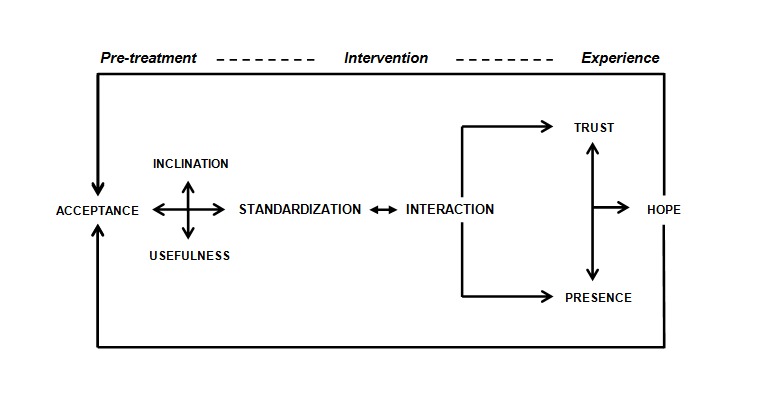
Engagement expectations regarding online psychotherapy for depression.

The patients, primary care physicians, and managers showed a series of expectations regarding engagement with the use of Web-based psychotherapy for the treatment of depression, which can be divided into three main groups: aspects related to pre-treatment conditions, such as their initial acceptance, their general inclination to make use of the treatment, and its perceived usefulness; aspects related to the particular characteristics of this type of intervention, such as its standardization and the processes of interacting with the program; and the representations made of what could be seen as the expected therapeutic experience, such as experiences of trust, presence, and hope. These groupings can be arranged according to the moment in the therapeutic process to which they correspond (Figure 1).

### Conceptualization of the Discourse

The general acceptance of Web-based psychotherapy for depression depended on prior conditions, such as the inclination to use new technologies, which in turn consisted of subtopics such as “skill level”, “reasons for use”, “available technical means”, and “dedication”. Acceptance also depended on its usefulness as an instrument for psychotherapy, and usefulness consisted of subtopics such as “facilitating communication” and “access to information”, which in the case of physicians took on the meaning of consulting the records or background of patients:

We have limited access to sources of information during a consultation. The computer is the tool that provides access to the most updated, complete, and verified information, depending on the platforms you consult. It’s an important tool for both receiving and conveying information. It also allows us to have the patient’s records online and on hand.

physician, female, 50 years old, high affinity

Whereas, for patients, it meant having the possibility of consulting information related to their conditions and its treatment:

The machine [sic] should explain the diagnosis of the disease or anomaly a patient has and quickly help to find solutions for daily life.

patient, male, 65 years old, high affinity

Other subtopics under usefulness were “adding convenience”, which for managers meant providing patient access to health care resources:

Maybe it will make professionals more accessible to users; it could become more convenient to avoid having to travel, and interfere less with people’s work because they can do it at home.

manager, female, 51 years old, high affinity

Whereas, patients understood it to mean the possibility of improving reminders for control of and compliance with the instructions given by the professional:

The program should have some mechanism to do online activities and revision; for example, what the doctor told me, to refresh the information so as not to forget it.

patient, female, 59 years old, low affinity

Finally, usefulness also included “enabling reflectiveness”, understood by patients to mean the opportunity to reflect on their dysfunctional situation:

Does the program have a written format? If that’s the case, writing means thinking and rethinking; it’s like a kind of meditation or awareness of the day, of how things are going, how they develop. It gives you perspective, and that’s positive.

patient, female, 52 years old, intermediate affinity

Physicians particularly focused on their awareness of diagnostic impressions resulting from explorations:

The advantage it may have is that you can prepare beforehand the information you’re going to introduce: the questions; results of a particular test; the answers; and you can leave a record of your recommendations, so that there are no misinterpretations. This encourages patients to elaborate on what took place during the consultation once outside, in a more relaxed atmosphere like that of their own homes.

physician, female, 42 years old, high affinity

Acceptance depended on the processes required to standardize the program, which consisted of the subtopics “learning process”, “definition of goals”, “complementary tool”, “prior dissemination”, “service offered” (particularly for managers), “professionals involved” (especially highlighted by physicians), “patients served”, and “activities to carry out”. The last was understood by patients according to their own needs:

I do yoga on Wednesday and I feel great. Maybe it could include relaxation techniques and different strategies for different states of mind; some days are better than others.

patient, male, 46 years old, intermediate affinity

Whereas physicians underscored the importance of being able to use their own paradigmatic coordinates:

In our specialty, we refer to those of a cognitive behavioral model. Other more introspective or psychodynamic models revolve around the doctor-patient relationship, which cannot be applied using technology.

physician, male, 51 years old, intermediate affinity

In turn, the necessary processes for standardization were associated with the idea of specific forms of interaction between the participants and the program. This idea of interaction combined subtopics such as “feeling of security”, “program universality”, “possibilities for expression”, “supervision by a therapist”, and “individualized attention for patients”. The last of these had special relevance for patients, in the sense of personal and close contact:

Rapport. You have to be on the same wavelength and make a connection. You need warmth—they’re personal matters—personal contact, personal proximity.

patient, male, 64 years old, intermediate affinity

For professionals, it was simply focused on the need to adapt to the individual characteristics of each patient:

If it were also a little... if the doctor could tailor the treatment a little and if it were based on a shared model of decision-making, with a certain proximity between the professional and patient.

physician, female, 54 years old, intermediate affinity

Finally, if these forms of individualized interaction, associated with the processes for standardization of the program, created expectations of a therapeutic experience based on trust and on presence, the discourse would pave the way to hope, which could lead to positive attitudes once again linked to acceptance as a necessary condition for engagement with the therapeutic process.

Table 3 shows the definitions of the topics and subtopics that comprise the conceptualization of the discourse presented above, and Multimedia Appendix 1 provides examples of participants’ responses to all of them.

**Table 3:** Conceptualization of discourse and definitions.

Topic/subtopic		Definition
**Acceptance**		
	Acceptance	Favorable attitude to therapeutic use of new technologies (NTs), with responsibility to accept the program with the necessary effort, recognizing its possible benefits.
**Inclination**		
	Skill level	Level of knowledge and skill when using NTs.
	Reasons for use	Reasons for the use of NTs, for work, leisure, etc.
	Available technical means	Available technical means and possibilities of updating them.
	Dedication	Time devoted NTs and form of use, taking into account the risks of patterns of abusive use in some individuals.
**Usefulness as an instrument for therapy**		
	Facilitating communication	NTs as alternative means of communicating and forming relationships with others, of keeping in contact, alerting, and giving notification of results.
	Access to information	Possibility of consulting background information and patient records (physicians) and important matters related to medical conditions and treatments (patients).
	Adding convenience	Solutions to problems with daily clinical practice, such as facilitating access (managers) or reminders for, control of, and compliance with instructions (patients)
	Enabling reflectiveness	Possibility of thinking/reconsidering through the use of writing, making possible a reflective attitude to their dysfunctional situation (patients), or of taking note of exploration and diagnostic impressions (physicians).
**Program standardization**		
	Learning process	Ease of learning the program and ease of use.
	Definition of goals	Structured definition of the goals to meet with the program and their timeframe.
	Complementary tool	Combined and complementary use of Web-based and traditional therapies, without the intention of replacing the presence of professionals.
	Prior dissemination	Dissemination of information about the program in order to overcome possible resistance to change based on old habits.
	Service offered	Implementation in health care delivery system, with a structure focused on a new model of health care, and with support from health care providers, IT experts, and administrative staff that allow referrals from primary care in coordination with mental health services.
	Professionals involved	Recruitment of professionals with a positive attitude toward NTs who have specific skills depending on the characteristics of this type of intervention.
	Patients served	Recruitment of patients according to their willingness to use NTs, low or mild severity of their disorder, and their demand for greater information or anonymity.
	Activities to carry out	Incorporation of modules that appeal to patients, arouse their interest, and meet their needs (patients), with real possibilities of implementation within paradigmatic coordinates (physicians).
**Interaction processes**		
	Feeling of security	Combination of safeguarding anonymity, data protection, and program quality.
	Program universality	Sharing experiences of illness within a group by means of supervised forums, owing to the general nature of dysfunction shared by all the patients.
	Possibilities for expression	Possibility of manifesting or expressing feelings and emotions through NTs.
	Supervision by a therapist	Appraisal of the evolution of the disorder throughout the therapeutic process (physicians).
	Individualized attention for patients	Personal, empathetic, sincere, and close contact (patients), with flexibility to be adapted to the individual characteristics of each patient (physicians).
**Trust**		Peace of mind when using the program in an intimate and participative way.
**Presence**		Support from and availability of a professional who listens and pays attention (patients).
**Hope**		An optimistic perspective for the outcome of the treatment.

### Participants’ Perspectives

In general, the participants similarly conceptualized their expectations regarding Web-based psychotherapy for depression, although the focus of their discourse offered differing perspectives depending on their position (Table 4). For example, the health professionals—physicians and managers—placed emphasis on the intervention processes from the perspective of standardization of the program. More specifically, the physicians centered their interest on the professionals involved, with the concern of whether this type of intervention could improve the current delivery of health care, but at the cost of increasing their already excessive workload. However, the discourse given by the managers was focused on the idea of the service provided, with interest shown in the cost-effectiveness of this resource, by means of an organization of work that would create a new dynamic for distributing patients, and in a way that would solve practical problems, such as reducing waiting lists. Finally, patients highlighted the intervention processes from the perspective of interaction with the program, through which they expected to receive quality, personal, and individualized service, which would offer them the possibility of safely sharing experiences with people in the same situation as theirs, and with an accessible professional who would be in charge of supervising the treatment.

The different perspectives expressed by the participants—physicians, managers, and patients—reflected their different concerns based on their position. Could a patient be placed in the program without increasing my workload? Would this program solve the problem of waiting lists without added expense? Would this program sufficiently attend to my particular condition? Each position raised the need to satisfy different requirements, and any of these could cause resistance to the introduction of this type of instrument into the health service.

**Table 4:** Participants’ discursive perspectives.

Participant	Focus of discourse	Quotes
Physicians	Professionals	“Maybe we could use a little help so as not to overload our schedules even more.” (physician, male, 45 years old, high affinity)
		“The design is relatively simple to imagine, but it means a change to our routine practice. How long will it take to train us to use and implement this program? Would we be released from our clinical practice for that time?” (physician, female, 54 years old, intermediate affinity)
		“Not all of us have the same technological know-how or motivation. Should all doctors use the tool or only the most qualified? Those of us who volunteer to participate could be released from our routine workload to devote ourselves more specifically to this matter”. (physician, female, 35 years old, high affinity)
		“Telemedicine platforms are already showing great improvement in places with dispersed populations, assisting with the organization of care and better communication between primary and specialist care services, which not only improves final care, but saves money.” (manager, female, 51 years old, high affinity)
Managers	Service	“The tool should speed up care processes, especially waiting lists and user access to resources, providing maximum efficacy with a small investment that is quickly justified.” (manager, male, 55 years old, intermediate affinity)
		“If we all save time with this tool, seeing more patients in distance mode and therefore freeing up our schedules, close, properly programmed, face-to-face monitoring could be carried out.” (manager, male, 50 years old, high affinity)
		“Of course, every patient is different, so if you decide to help them, first you have to know their whole story.” (patient, male, 46 years old, intermediate affinity)
Patients	Individualization	“Depending on the patient, on the state they’re in, there can be interventions that are for everybody. But some patients, given their particular evolution, will need more personalized care.” (patient, female, 52 years old, intermediate affinity)
		“Therapy has to be something that is very personalized, with the possibility of asking and speaking about other subjects...” (patient, female, 59 years old, low affinity)

## Discussion

### Principal Findings

Our results show how important it is for primary care physicians that the implementation of Web-based psychotherapy programs does not mean an added workload for them; rather, this should be reduced. They also show that the main concern for health care management professionals is to offer greater accessibility to services, and to improve their cost-effectiveness. Patients expect to receive individualized care, which requires investment in a certain level of resources, workload for professionals, and infrastructure in order to meet their needs. These particular viewpoints reflect different expectations, which taken individually, seem to respond to conflicting needs and interests. In order to overcome any possible resistance simultaneously, we have to find common ground. One starting point in this regard could be the optimization of the processes for management and patient flow in an integrated model, which has proven to be very useful for balancing the burdens of care delivery [58].

Depressive disorder is currently one of the greatest challenges for public health systems. In its mild and moderate forms, it responds satisfactorily to different types of psychotherapeutic approaches [59], such as Web-based cognitive behavioral therapy, including those with little or no support [60,61]. This type of treatment could become an alternative for public health systems to facilitate patients’ acceptance and access to care [62], adding efficacy to early interventions [22,40,41]. Forecasts show that the dissemination and use of Web-based psychotherapy will grow exponentially in the coming decade [63]. In this context, it is necessary to establish procedures for the implementation of these type of programs that meet the many varied expectations placed on them by all participants in the therapeutic process, whether patients or health professionals.

A fundamental aspect in this regard would be to determine the level of intensity of the required supervision. The Spanish health care system treats all individuals equally, which means that the referral process for patients is not a fast and smooth one. In an attempt to optimize the therapeutic process, patients should be and feel cared for, without overloading professionals, but while reducing costs and waiting times [64]. In order to do this, it would be necessary to perform the specific task of disseminating the program, which would allow the patients most likely to benefit from this type of treatment to be identified and chosen, without losing sight of the factors that would enhance compliance. Those patients with symptoms of mild severity are seen as good candidates, as well as those who take responsibility for the treatment and who attribute success to themselves, focusing on action [65-67]. According to our results, all participants would show moderate severity at most, with an attitude of general acceptance toward Web-based therapy and a certain inclination for the use of new technologies, as has been seen in other works [68], but most of all, they have to understand their possible usefulness in the specific field of psychotherapy.

Both patients and health professionals have to share a certain willingness to use new technologies [68], must have a certain level of skill in their use, and need to have the necessary infrastructure [69], which is particularly important when we encounter difficult-to-access settings, or in cases with scarcity of training and resources [70,71]. All participants need to perceive the possibilities offered by Web-based psychotherapy as useful, with regard to convenience, time, and place of use, and value the reduction of transport costs, and the possibilities for communication, reflectiveness, memory, and information that it brings, while giving patients an active role and enabling its use whenever they want [72]. Nevertheless, all participants also highlighted the importance of not falling into patterns of abuse. In other words, the use of Web-based psychotherapy for the treatment of depression should provide everybody involved with a certain sense of efficacy and comfort [69]. This is how depression can become easier to handle, probably through patients’ actual perception of their own self-efficacy.

We have seen that the discourse of professionals basically revolves around the difficulties of attempts at standardization, which is necessary in order to provide institutional support for Web-based therapy. This is a reference to the general culture in which health services are immersed [73]. This culture is present in questions such as learning processes, definition of goals to be met and activities to carry out, and in relation to the professionals involved and the services provided. That is to say, the group of health professionals—physicians and managers—are interested in the implementation of the program as a tool for clinical intervention, with the expectation that the needs of their users/patients have to be met, but also those of the professionals involved, implying possible strategies for promotion and improvement in quality and efficiency. We have thus seen how health care workers highlight the importance of standardization processes for Web-based treatments as part of an integrated service model, which is in agreement with the results of other studies [68-74].

In general, health professionals have positive attitudes to Web-based treatments [75,76], and these positive attitudes tend to be associated with an open mind and availability to new treatments, the convenience that computers offer, and easy access to technology [69], which is in line with our results. Nevertheless, therapists have also been said to express doubts as to whether new technologies produce substantial improvements, and although they are generally open to incorporating Web-based psychotherapy into their clinical practice, they show doubt depending on their skill, and out of the belief that this type of therapy may harm their relationship with patients [69]. In this regard, our study has pointed out, through the discourse of both professionals and patients, that therapeutic interaction by computer should not completely replace human contact, which underscores the limits to the application of indiscriminate Web-based psychotherapy.

Web-based therapeutic interventions are rarely conducted without face-to-face contact at some point in the process [27,77,78]. The presence of a therapist is key to guiding patients, given that this benefits the treatment, and supervision of the therapeutic process is associated with achieving positive results, while withdrawals from treatment increase when there is not sufficient contact [27,30,79]. On the contrary, adding contacts may improve efficacy, probably as a result of the trust created in the process [80]. When we asked patients about the possibilities of Web-based psychotherapy, we immediately saw the emergence of thematic content that was linked to the need to construct a therapeutic alliance as a vector that produces the hope of a positive outcome [81-83]. In fact, it is the base on which all psychotherapy is constructed, as a relationship of help [84,85]. Another important aspect to take into account is the necessary confidentiality of the entire process, for the security and anonymity that both patients and professionals require [68], and given that this could help to prevent stigmas [65], if clinical symptoms are able to be resolved at the primary health care level, providing solutions without the need for involving mental health units [86].

In general, the context for applying these results does not seem to be logically restricted to the particular characteristics of the place where the study was conducted. Instead, it points out the possible general characteristics and interests of the different participants. While all of the participants resided in only one region of Spain, it is true that Spanish primary care practices on the whole are currently a setting with a very high care delivery burden [87], and patient profiles also show a certain tendency toward personal contact in visits, as occurs in the majority of countries studied [88]. For this reason, our results seem to be fairly reliable. Nonetheless, it would be logical to propose future studies that make use of our procedures in other countries, in order to verify the extent to which our conclusions can be generalized in different populations, or whether they are in fact the specific product of the studied population. The fact that the interviewer and analysis team were mental health specialists, psychologists, and psychiatrists should not be overlooked, and they may therefore have focused the framework of discourse generation and the creation of emerging categories toward topics of special interest in this field of knowledge. We would add that the randomized controlled study conducted parallel to this work, although with different participants, may have exerted a certain influence on the processes of analysis and interpretation of the data.

### Conclusions

This study shows how patients, physicians, and managers see the possibility of carrying out Web-based psychotherapy for the treatment of depression revolve around two attractors: the need for individualized interaction, which enables the establishment of a therapeutic alliance, and the processes for standardizing the program, which would prevent professional overloading while optimizing cost-effectiveness. In order to find a balance between both expectations, Web-based psychotherapy should be understood as a complementary intervention, which allows the amount of human contact to be reduced, but without completely eliminating it. This type of intervention provides a way of treating depression, which would permit more patients to be supervised with fewer visits, offering a solution to the problem of waiting lists, and without being an additional burden for health professionals. Research shows that Web-based psychotherapy as an approach to treating depression is in its infancy as a therapeutic option. In order to facilitate its implementation in primary care, we should be aware of the expectations of all participants by designing effective programs that are able to offer patients individualized treatment by means of standard interventions in the different contexts. Incorporating Web-based therapy into health systems harmoniously will show its value in improving delivery of care [89], by facilitating access to resources and improving working conditions for professionals.

## Multimedia Appendix 1

Participants’ responses to subtopics.
