# Supplementary material for: Expectations Among Patients and Health Professionals Regarding Web-Based Interventions for Depression in Primary Care: A Qualitative Study
Source: J Med Internet Res. 2015 Mar 10;17(3):e67. doi: 10.2196/jmir.3985 (PMC4376189; doi:10.2196/jmir.3985)
Supplement: Supplementary file 1 [file jmir_v17i3e67_app1.pdf]

Multimedia Appendix 1: Participants' responses to subtopics

**Acceptance**	
Acceptance	*"It's an innovative idea and I'm sure it will provide a good outcome, because patients naturally have more access to networks every day. So, as another tool, of course it can be positive."* (physician, female, 30 years old, high affinity)
**Inclination**	
Skill level	*"Well, I can use a computer more or less okay, not as good as my daughter, for instance... but I don't have any problems."* (patient, female, 51 years old, low affinity)
Reasons for use	*"I mainly use it for work and for socializing."* (patient, female, 32 years old, high affinity)
Available technical means	*"Well, I have a computer at home, what else? Of course I also have a smartphone."* (patient, male, 64 years old, high affinity)
Dedication	*"At work, not all day, but if you're checking your emails and looking for information. Afterwards at home… then its every day, browsing, checking emails, and reading news of interest (…) I always draw a line, of course… You know, technology can take over… you can spend all the time you want with it."* (patient, female 61 years old, high affinity)
**Usefulness as an instrument for therapy**	
Facilitating communication	*"At times, especially for contact between people who live very far away, which is what happens in rural areas. I see it as very useful."* (manager, male, 55 years old, intermediate affinity)
Access to information	*"We have limited access to sources of information during a consultation. The computer is the tool that provides access to the most updated, complete, and verified information, depending on the platforms you consult. It's an important tool for both receiving and conveying information. It also allows us to have the patient's records online and on hand."* (physician, female, 50 years old, high affinity)
	"The machine [sic] should explain the diagnosis of the disease or anomaly a patient has and quickly help to find solutions for daily life." (patient, male, 65 years old, high affinity)

Adding convenience	*"Maybe it will make professionals more accessible to users; it could become more convenient to avoid having to travel, and interfere less with people's work because they can do it at home."*<br>(manager, female, 51 years old, high affinity)<br><br>*"The program should have some mechanism to do online activities and revision; for example, what the doctor told me, to refresh the information so as not to forget it."*<br>(patient, female, 59 years old, low affinity)
Enabling reflectiveness	*"Does the program have a written format? If that's the case, writing means thinking and rethinking; it's like a kind of meditation or awareness of the day, of how things are going, how they develop. It gives you perspective, and that's positive."*<br>(patient, female, 52 years old, intermediate affinity)<br>*"The advantage it may have is that you can prepare beforehand the information you're going to introduce: the questions; results of a particular test; the answers; and you can leave a record of your recommendations, so that there are no misinterpretations. This encourages patients to elaborate on what took place during the consultation once outside, in a more relaxed atmosphere like that of their own homes."*<br>(physician, female, 42 years old, high affinity)
**Program standardization**	
Complementary tool	*"It could be like a support tool for certain things, like support and reinforcement for face-to-face therapies. I think it' very useful, but as a support and reinforcement."*<br>(patient, female, 59 years old, low affinity)
Prior dissemination	*"Professionals and patients first have to know what it is that we're doing, why we're doing it and the specific goals, and how it can be beneficial and useful for them. We have to convey that we are making new technologies available to them... I think that a lot of the reticence we encounter for the new is often because we don't explain things enough."*<br>(manager, male, 50 years old, high affinity)
Service offered	*"If it's standardized, if all the IT systems are unified, then fantastic. We should all use the same tools to prescribe treatments, guide and refer cases on, so that the*

	system and the new tool work smoothly." (physician, male, 48 years old, low affinity)
Professionals involved	*"It would be important to choose suitable professionals based on their interest, skill, and availability to carry out the supervised part of the tool."* (physician, female, 35 years old, high affinity)
Patients served	*"What type of depressive disorders has to be defined... It could be useful for mild cases, but it wouldn't even occur to me to use it with a severe disorder: that's a tall order, a very serious illness."* (manager, female, 51 years old, high affinity)
Learning process	*"it should have simple operating procedures that allow you to get the most out of the possibilities of the program with little training."* (patient, male, 46 years old, low affinity)
Definition of goals	*"It would have to be highly defined. With clear rules and guidelines."* (patient, male, 64 years old, intermediate affinity)
Activities to carry out	*"I do yoga on Wednesday and I feel great. Maybe it could include relaxation techniques and different strategies for different states of mind; some days are better than others."* (patient, male, 46 years old, intermediate affinity) *"In our specialty, we refer to those of a cognitive behavioral model. Other more introspective or psychodynamic models revolve around the doctor-patient relationship, which cannot be applied using technology."* (physician, male, 51 years old, intermediate affinity)
**Interaction processes**	
Feeling of security	*"It has to work correctly, and not crash. The platform has to keep patient information reliably, and guarantee its proper protection."* (physician, female, 35 years old, high affinity)
Program universality	*"Sharing experiences with other people who are going through what you are would be very positive. They can understand you, and if you are feeling low and they are better, then can cheer you up and help."* (patient, female, 45 years old, low affinity)
Possibilities for expression	*"Probably for those things that are of a much more personal, affectionate, emotional nature, it's difficult to replace a personal and face-to-face interview, although it's also true that there are times where a certain distance and anonymity makes it*

	easier to express certain subjects.”<br>(manager, male, 55 years old, intermediate affinity)
Supervision by a therapist	*“I think it's a very good initiative, as long as there's a professional behind it evaluating and following the clinical evolution.”*<br>(physician, male, 48 years old, low affinity)
Individualized attention for patients	*“Rapport. You have to be on the same wavelength and make a connection. You need warmth – they’re personal matters – personal contact personal, proximity.”*<br>(patient, male, 64 years old, intermediate affinity)<br><br>*“If it were also a little... if the doctor could tailor the treatment a little and if it were based on a shared model of decision-making, with a certain proximity between the professional and patient”.*<br>(physician, female, 54 years old, intermediate affinity)
**Trust**	
Trust	*“Using a tool like this calmly is closely related to the trust the patient feels; and this trust is built up over time, the time you've been with that patient"*<br>(physician, female, 54 years old, intermediate affinity)
**Presence**	
Presence	*“If something comes up at any time, you need to know that the professional is there and can sort you out, because you can tell when you have someone who's qualified and who's concerned”*<br>(patient, female, 59 years old, low affinity)
**Hope**	
Hope	*“It has something to do with things that we overlook in the practice, but patients sometimes show their trust, and sort of take a huge step forward and think that what you're offering them will be good for them”*<br>(physician, female, 35 years old, high affinity)
